# Faecal Microbiota in Patients with Neurogenic Bowel Dysfunction and Spinal Cord Injury or Multiple Sclerosis—A Systematic Review

**DOI:** 10.3390/jcm10081598

**Published:** 2021-04-09

**Authors:** Willemijn Faber, Janneke Stolwijk-Swuste, Florian van Ginkel, Janneke Nachtegaal, Erwin Zoetendal, Renate Winkels, Ben Witteman

**Affiliations:** 1 Heliomare Rehabilitation Centre, 1949 EC Wijk aan Zee, The Netherlands; 2 Center of Excellence for Rehabilitation Medicine, Brain Center Rudolf Magnus, University Medical Center Utrecht and De Hoogstraat Rehabilitation, Utrecht University, 3583 TM Utrecht, The Netherlands; j.stolwijk@dehoogstraat.nl; 3 Faculty of Medicine, Utrecht University, 3584 CG Utrecht, The Netherlands; f.vanginkel@students.uu.nl; 4 Heliomare Rehabilitation Center, Department of Research & Development, 1949 EC Wijk aan Zee, The Netherlands; J.Nachtegaal@heliomare.nl; 5 Laboratory of Microbiology, Wageningen University and Research, Wageningen University, 6708 PB Wageningen, The Netherlands; erwin.zoetendal@wur.nl; 6 Division of Human Nutrition and health, Wageningen University and Research, Wageningen University, 6708 PB Wageningen, The Netherlands; renate.winkels@wur.nl (R.W.); ben.witteman@wur.nl (B.W.)

**Keywords:** spinal cord injury, multiple sclerosis, neurogenic bowel dysfunction, gut microbiota

## Abstract

Background: Neurogenic bowel dysfunction (NBD) frequently occurs in patients with spinal cord injury (SCI) and multiple sclerosis (MS) with comparable symptoms and is often difficult to treat. It has been suggested the gut microbiota might influence the course of NBD. We systematically reviewed the literature on the composition of the gut microbiota in SCI and MS, and the possible role of neurogenic bowel function, diet and antibiotic use. Methods: A systematic search was conducted in PubMed and Embase, which retrieved studies on the gut microbiota in SCI and MS. The Newcastle–Ottawa Quality Assessment Scale (NOS) was used to assess methodological quality. Results: We retrieved fourteen papers (four on SCI, ten on MS), describing the results of a total of 479 patients. The number of patients per study varied from 13 to 89 with an average of 34. Thirteen papers were observational studies and one study was an intervention study. The studies were case control studies in which the gut microbiota composition was determined by 16S rRNA gene sequencing. The methodological quality of the studies was mostly rated to be moderate. Results of two studies suggested that alpha diversity in chronic SCI patients is lower compared to healthy controls (HC), whereas results from five studies suggest that the alpha diversity of MS patients is similar compared to healthy subjects. The taxonomic changes in MS and SCI studies are diverse. Most studies did not account for possible confounding by diet, antibiotic use and bowel function. Conclusion: Based on these 14 papers, we cannot draw strong conclusions on the composition of the gut microbiota in SCI and MS patients. Putatively, alpha diversity in chronic SCI patients may be lower compared to healthy controls, while in MS patients, alpha diversity may be similar or lower compared to healthy controls. Future studies should provide a more detailed description of clinical characteristics of participants and of diet, antibiotic use and bowel function in order to make valid inferences on changes in gut microbiota and the possible role of diet, antibiotic use and bowel function in those changes.

## 1. Introduction

Multiple sclerosis (MS) has been estimated to affect 2.3 million people globally and prevalence of spinal cord injury (SCI) ranges from 223 to 755 per million people globally [1,2]. Both numbers are increasing each year. One of the most often reported secondary complications in individuals with SCI is neurogenic bowel dysfunction (NBD) [3]. NBD is a severe disabling impairment and can be caused by SCI and MS. It is defined as a colonic and/or anorectal dysfunction resulting from a lack of central nervous control [4]. SCI and MS patients often suffer from the same symptoms and the etiology, dysfunction of the spinal cord, is compatible. Bowel management can reduce the impact on a person’s quality of life (QOL) and can prevent faecal incontinence and constipation [5,6]. Current guidelines refer to a stepped-up pyramid tool for bowel management in individuals with MS and SCI [7]. The first step in the pyramid is optimizing dietary and fluid adjustments or the use of stool modulating agents (e.g., stool softeners, stimulant laxatives and bulking agents) [8,9]. The next steps are the use of more invasive techniques, such as the perianal/rectal stimulation technique, a manual removal of faeces or transanal irrigation [10]. Finally, the implantation of electrical stimulation systems, antegrade colonic enemas or the formation of a bowel stoma are all possible treatment options if problems persist.

Of the MS patients, 39–73% report neurogenic bowel problems [11]. There appears to be a correlation between bowel problems and the Expanded Disability Status Scale (EDSS) and disease duration, but not the type of MS [12,13,14,15,16]. Surprisingly, MS patients with a short period of time since onset and a low disability can also have bowel problems, with severe constipation having been reported as the first symptom of MS [17]. MS patients score their bowel problems as the third-most bothersome symptom. These problems are a major cause of not being able to participate in society and work and account for a significant part of the daily routine [11].

From research done in the SCI rehabilitation centres in the Netherlands, we know that 31% of the sub-acute SCI patients are not satisfied with their bowel functions at the moment of discharge from their first inpatient rehabilitation. NBD can result in faecal incontinence, abdominal bloating, and constipation [5,6]. In the chronic phase, this percentage increases up to 80% [4]. In a survey among 1334 people with SCI, for instance, 39% reported constipation, 36% haemorrhoids, and 31% abdominal distension [4]. Other issues that were reported included diarrhoea and incontinence [8]. NBD following SCI has a huge impact on the QOL [8]. In people with faecal incontinence, 62% reported a negative effect on the QOL compared to 8% in controls [18]. A questionnaire completed by members of the Dutch Spinal Cord Injury Patient Society, showed bowel problems as the second most important topic that, according to patients, should be studied more.

It is hard to achieve adequate bowel management in NBD as bowel management is influenced by many factors such as diet, level of mobility or pharmacological treatment [4]. One of the factors could be the gut microbiota. There is some evidence that alteration of the gut microbiota could result in better bowel function in the healthy population, patients with Irritable Bowel Syndrome or SCI [3,19,20].

The composition and activity of the gut microbiota co-develop with the host from birth and is subject to a complex interplay. There are numerous host factors, such as age, gender, and ethnicity, as well as environmental factors related to our lifestyle that can influence the gut microbiota [21,22,23,24].

A large Flemish/Dutch study on gut microbiota variation in the average, healthy population showed that of all measured factors, stool consistency has the largest effect size [25]. The increase of transit time, independent of other factors, may affect the composition and metabolism of the gut microbiota as well. The transit time is one of the factors that explain some of the modifications seen in the gut microbiota of the elderly, as well as in patients with slow transit time [26]. Several studies with SCI patients show longer colon transit time compared to uncompromised subjects [27].

Alterations in diet, primarily influenced by the consumption of dietary fiber from fruits, vegetables, and other plant components, have been associated with changes in the gut microbiota. It has been reported that even a short-term dietary shift can significantly change gut microbiota [28]. NBD and altered colonic transit time in SCI and MS patients might lead to a change in the composition of the gut microbiota that might be influenced by a diet change. Therefore, the first step in bowel management in SCI and MS patients with NBD could be a specific diet to target the gut microbiota in order to improve the intestinal complications in SCI and MS patients.

In addition to the impact of diet, treatment with most antibiotics, especially broad-spectrum antibiotics, have also been shown to affect the gut microbiota composition. Antibiotic therapies may affect not only the target microorganisms but also the host-associated microbial communities, particularly those in the intestine [29]. In MS and SCI patients, neurogenic lower urinary tract dysfunction, respiratory and skin problems frequently occur [30] and hence this population is at risk of developing infections that often require antibiotic treatment [31,32]. Therefore, they might also be at risk of altered gut microbiota composition.

The following research questions for this systematic review are based on the possible NBD of SCI and MS patients, their frequent use of antibiotics and the distinct impact of diet: What is the difference in the composition of the gut microbiota, with focus on bacteria, of patients with SCI or MS compared to HC? What is the possible role of neurogenic bowel function, diet and antibiotic use on the composition of the gut microbiota? 

## 2. Methods

### 2.1. Information Sources

**Figure 1:**
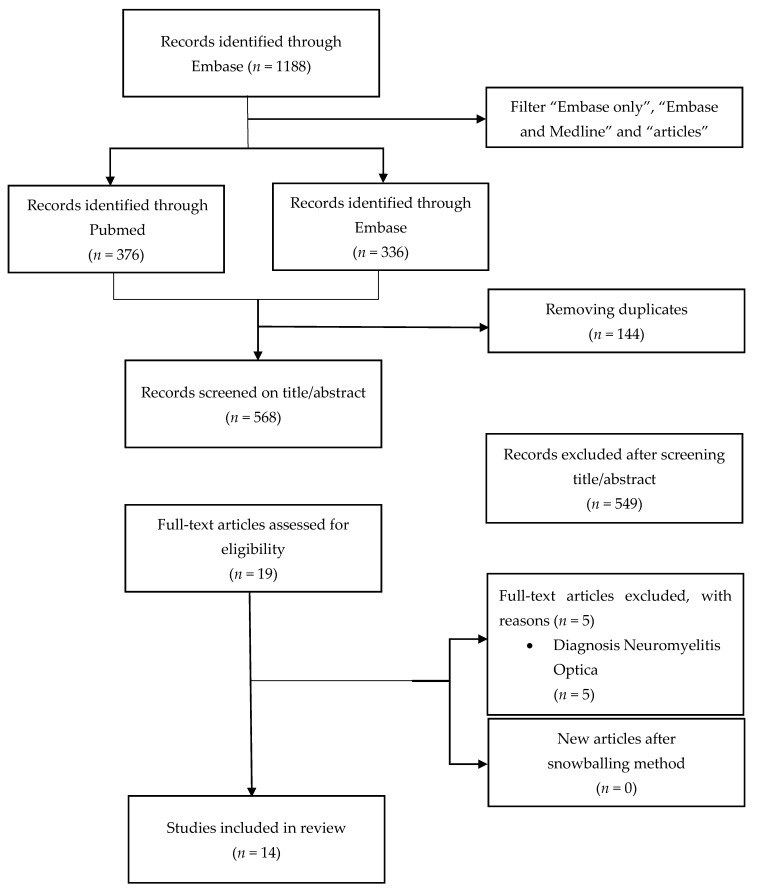
Flowchart of studies through the screening process.

This review was performed in accordance with the Preferred Reporting Items for Systematic Reviews and Meta-Analyses (PRISMA) [33]. Studies were identified by searching the National Library of Medicine (PubMed) and Excerpta Medica (Embase) for available studies on the gut microbiota of patients with NBD due to SCI or MS. The search was performed on 8 July 2020. Figure 1 shows the flowchart of studies through the screening process. The search terms consisted of the following keywords including Medical Subject Headings (MeSH)terms, synonyms and acronyms: “multiple sclerosis”, “spinal cord injury”, “gastrointestinal microbiome”, “dysbiosis” and “stool sample”. The full syntax can be found in Table A1.

### 2.2. Eligibility Criteria

Two independent reviewers (WF and FG) screened the studies on eligibility for inclusion in the review using Rayyan [34]. Firstly, studies were screened by title to exclude studies that clearly did not meet the eligibility criteria. Then, the abstracts of the remaining studies were screened and finally, the full-text articles were screened. On top of the database searches, after screening the abstracts, the reference lists were also checked to prevent missing relevant studies. Differences between the reviewers in agreement to include a study were assessed at both stages and were discussed to reach consensus.

Studies that met the following criteria were included:

- Study on the gut microbiota of patients with SCI or MS.
- Study included a group of HC.
- Participants were aged 18 years and older.
- Gut microbiota composition was determined by 16S rRNA gene sequencing.
- Published as full-text article in English in a peer-reviewed journal.

Studies which focused on Neuromyelitis Optica were excluded.

### 2.3. Data Extraction and Outcome Measures

WF extracted the data from the full-text articles, which was checked by a second reviewer (JN).

Extracted data included: (1) authors and publication year, (2) objective of the study, (3) characteristics of the included study sample (sample size, mean age, disease characteristics) (4) study design (including number of faecal samples taken), (5) outcome variables and potential confounding factors including use of antibiotics, bowel function, and diet, (6) results.

The main outcome was the difference between the composition of the gut microbiota of patients with NBD due to SCI or MS and that of HC. Differences in gut microbiota are defined as differences in diversity and taxonomic differences. Alpha diversity provides a measure of the variety of the species represented within the sample.

In addition, an evaluation took place of which studies took into account the role of antibiotic use, diet, and bowel function on the gut microbiota.

### 2.4. Quality Assessment

The quality of the included studies was assessed with the Newcastle–Ottawa Quality Assessment Scale (NOS) [35]. The NOS contains eight categories in the selection of cases and controls, comparability of the groups, and establishment of outcome. A study can be awarded a maximum of one star for each numbered item within the Selection and Exposure categories, a maximum of two stars can be given for comparability. A score of 0–3 points is defined as a study of low quality, a score of 4–6 points represents a moderate quality study, and studies with 7–8 points are studies of high quality. The quality of the included studies was independently assessed by WF and JN. Both reviewers checked the article together in the event of discrepancies in scores in order to reach consensus on the score.

## 3. Results

### 3.1. Literature Search

The PubMed databank was searched with aforementioned terms. As a result, we came up with 376 articles. We also searched through Embase, which resulted in 1188 articles, and subsequently filtering on “Embase only” or “Embase and Medline” reduced this to 336 articles. After removing the duplicates from the total of 712 articles, we identified 568 articles that fulfilled the inclusion criteria.

Subsequent selection based on content described in the abstracts resulted in 19 articles that both reviewers agreed on their inclusion. We also checked the references lists but did not find any extra articles. Then, after reading the full articles, we excluded another five articles because of Neuromyelitis Optica diagnosis of the patients. In this category of MS patients, bowel problems are not very common. In total, we found four articles on SCI and ten articles on MS (Figure 1).

### 3.2. Description of Included Studies

Twelve papers were observational, cross-sectional studies; one study was an observational longitudinal study [36] and one study was an interventional, longitudinal study [37]. In Table 1, we included a description of the included studies looking at sample size, disease characteristics, HC characteristics, mean age and number of faecal samples. Most studies had small sample sizes, varying between 13 and 89 patients. The number of HC varied between 14 and 165. The age of most patients and HC was between 30 and 40 years. In the four SCI articles [38,39,40,41], all studies described how long the injury existed. In the MS articles, some described the time since diagnosis but did not correct this for the outcomes. All MS articles included if their patients suffered from Relapsing Remitting MS (RRMS) or Primary Progressive MS (PPMS). Only some described if their patients were in an active disease state or in remission.

Twelve studies looked at just one faecal sample. One study looked at two samples of all the participants within a two-month interval. Finally, one study looked at samples of the HC every two weeks.

The recruitment of HC was different in every study. In seven studies, there were no specific descriptions of HC recruitment [39,40,41,42,43,44,45]. In three studies, HC were recruited from databases (Metabolic Department University Hospital Brussels [46], Norwegian Bone Marrow Donor Registry [37], Brigham and Women’s Hospital PhenoGenetic project [47]). In four studies, HC were recruited from hospital staff or students (Hospital Brussels (para)medical staff [46], University of Manitoba Health Sciences Centre [36], Turkish hospital employees [38], Azabu University [48]). In one study, family members were recruited [49] and in one study, the participants’ proxies were included as HC [46].

In four studies, HC and patients are matched for age [39,42,46,47]. In four studies, they were matched for geographical region [37,46,47,49]. Seven articles [36,40,41,42,45,47,49] were matched for medical history, including former diseases and medical conditions. There was only one study that matched for Body Mass Index [46]. The exclusion criteria for patients and HC within a study were mostly the same.

All studies determined the gut microbiota composition by 16S rRNA gene sequencing. Not all studies collected the faeces samples in the same way. Most samples were collected by participants at home, whilst some were collected at the hospital [38,40,41]. There were different kits and different storage temperatures. All samples in the articles were stored at −80 °C, with the exception of two articles, which were stored at −70 °C [42,43]. For DNA extraction, different kits were used. There were also differences in the targeted variable (V) region of the 16 rRNA. Six studies targeted V4 ([36,38,39,43,46,49]), four studies V3–V4 ([37,40,41,44]), three studies V3-V5 ([42,45,47]) and one study targeted V1–V2 ([48]). All of these methodological differences are big confounders, hampering a detailed comparative gut microbiota analysis between the different studies.

### 3.3. Quality Assessment within Studies

We used the Newcastle–Ottawa Quality Assessment Scale to assess the methodological quality of the case-control studies in this systematic review (Table A2. According to this scale, we did not find an article of low quality (0–3 points). Twelve articles were of moderate quality (4–6 points), with most studies (seven in total) scoring five points. There were only two articles of high quality: one article [41] with seven points and one article [49] with eight points. When we compared these articles, we did not find the same outcomes. Both articles excluded antibiotic use before the start of the study. But in none of these articles were the participants put on the same diet. Because none of the articles scored as low quality, we did not exclude any articles after completing this scale. The conclusion could be that the NOS is not specific enough, because the great majority scored moderate. On the category “comparability”, only two factors can be scored. In gut microbiota studies this might not be enough for comparability of cases and controls.

### 3.4. Alpha Diversity

When comparing the alpha diversity between groups of participants in the 14 publications, we found that six articles [36,40,41,43,46,48] showed a lower alpha diversity of bacteria in SCI and MS compared to HC, five articles [37,42,45,47,49] showed a comparable alpha diversity, while two articles [39,44] showed a higher alpha diversity. In one article there was no conclusion about alpha diversity [38] (Table 2).

When we looked at the SCI and MS group separately, we found in two articles [40,41] a lower alpha diversity in the SCI group compared to HC. In one article [39], there was a higher alpha diversity. In this last article patients had an acute spinal cord injury.

In the MS group, we found in five articles [37,42,45,47,49] a similar alpha diversity between MS and HC. Four articles [36,43,46,48] found a lower alpha diversity in MS compared to HC. In one study [44], a higher alpha diversity in MS compared to HC was found. This last study only had four stars on the NOS, which is the lowest score out of the fourteen articles (Table A2). In one article [46] a downward trend was found in alpha diversity from benign, active untreated MS to RRMS treated with interferon and untreated RRMS during relapse.

In conclusion, there is not an overall outcome that is unambiguous. However, there seems to be a lower or comparable alpha diversity in patients compared to HC.

### 3.5. Taxonomic Differences

Overall, all studies compared and contrasted gut microbiota composition at various levels and depth of analyses, but only some of them reported beta diversity observations. When looking at specific taxonomic differences in the respective articles, we did not find uniform observations between the studies. At the phylum level, however, we observed that Firmicutes and Bacteroidetes were the most dominant in all studies, the variation between studies is large and independent of the health status of the individual. Both lower and higher relative abundances of these phyla were observed in SCI and MS patients compared to HC. In five studies [36,39,40,45,49] we came across a higher relative abundance of Firmicutes and in four studies [41,42,43,46] a higher relative abundance of Bacteroidetes. Not surprisingly, higher taxonomic resolution up to genus level did not reveal consistent differences when comparing MS and SCI patients to HC. We speculate that these inconsistent observations are not only due to subject-specificity of the gut microbiota composition, but also to the result of many confounders between the studies (as will be discussed in the next section) that hamper a detailed comparison.

### 3.6. Variation in Design and Methodology between Studies

When comparing the different articles, we discovered differences between participant selection, the method of stool storage, DNA isolation and 16S rRNA gene sequencing (Table 1). There were different stool collection methods, storage temperatures and DNA extraction kits. Because of the variability across studies listed in Table 1, it is possible that the results may differ just because of the discrepancies in the above-mentioned topics. That is why in-depth comparison between the studies is hampered.

There were also different targeting regions of the bacterial 16S rRNA gene (Table 1). The chosen targeted 16S rRNA gene region and primers to use for amplification can also have a major impact on depth of taxonomic resolution for classification and overall gut microbiota profiles [50]. When we compared the six articles [36,38,39,43,46,49] with V4 being the targeted 16S rRNA gene region, in three of them [36,39,49] we found comparability with a higher relative abundance of the genus *Clostridium* (Phylum Firmicutes) in patients compared to HC. When we compared the four articles [36,43,46,49] with MS subjects and V4 being the targeted 16S rRNA gene region, we found in two articles [43,46] a similarity of a higher relative abundance of the genus *Bacteroides* (Phylum Bacteroidetes). When we compared the four articles [37,40,41,44], with V3–V4 being the targeted gene region, we found in two articles [37,40], a lower relative abundance of the genus *Faecalibacterium* (Phylum Firmicutes) in patients compared to HC. Furthermore, in two articles [40,44], we observed a higher relative abundance of Phylum Verrucomicrobia. When we compared the two articles [37,44] with MS patients and V3–V4 being the targeted gene region, we did not find uniform taxonomic differences between MS patients and HC. When we compared the three articles [42,45,47], with V3–V5 being the targeted gene region, we found in two articles [42,45] a higher relative abundance of Phylum Firmicutes and *Genus Dorea* (Phylum Firmicutes) in patients compared to HC. Overall, these observations indicate that the targeted 16S rRNA gene region impacts the findings of the different studies.

We also found variability between the cases and controls recruited in the different studies. In only four articles [39,42,46,47] participants were age-matched. In three articles [37,46,49] participants lived in the same geographical region. In seven articles [36,40,41,42,45,47,49], participants are matched for (part of their) medical history.

In light of our research question, we were especially interested in bowel function, diet and antibiotic use (Table 3).

Four articles [37,40,41,46] scored the bowel function of their participants. Only one article [41] collected NBD symptom dates in their patients and formed subgroups. They divided their patients into a “with constipation” group or “without constipation” group; they also formed a “bloating” and a “without bloating” group. The constipation group showed a higher relative abundance of the genus *Bifidobacterium* (Phylum Actinobacteria), the bloating group showed a higher number of the genus *Megamonas* (Phylum Firmicutes) and the without bloating group showed a higher number of the genus *Alistipes*.(Phylum Bacteroidetes). This specific article also gave their participants the same hospital food and excluded antibiotics.

Four articles [37,46,47,49] collected dietary intake data using a dietary survey, but provided only limited information about the exact method and findings, apart from one study [49], that concluded that yoghurt intake did not influence alpha diversity. Three studies [38,40,41] gave their participants the same hospital food (not further specified) for a certain period, prior to faeces collection. In two of these articles [40,41], a lower number of Phylum Firmicutes in patients compared to HC became apparent. In all three articles, we found a lower number of the genera *Megamonas* and *Dialister* (both Phylum Firmicutes) in patients compared to HC.

All studies but two [44,45] excluded antibiotic use before faeces collection. There were a lot of differences in the antibiotic exclusion period. We looked at the four articles [36,43,47,49] that excluded antibiotics for the longest period: more than eight weeks. In two of these studies [36,49], a higher number of the genus *Clostridium* (Phylum Firmicutes) was found in patients compared to HC. However, in a third study [43], a lower number of *Clostridium* in patients compared to HC was discovered. In this last study, the period without antibiotics was longer than the two studies with a higher number of *Clostridium*. The study [47] with the longest period without antibiotics (6 months) showed a higher number of phylum Verrucomicrobia and genus *Akkermansia* in patients compared to HC.

## 4. Discussion

Studies in the field of gut microbiota analysis are always difficult to perform because of general limitations. The composition is subject to a complex interplay and there are many factors that can influence the gut microbiota.

Our systematic literature review retrieved fourteen studies. Based on those studies, we cannot draw strong conclusions on differences between SCI or MS patients and HC about composition of the gut microbiota. Putatively, the chronic SCI group may have a lower alpha diversity compared to HC, while there are also some indications that the MS group shows mainly a compatible or a lower alpha diversity compared to HC. Taxonomic differences in both groups are too diverse to draw strong conclusions. The limited information about dietary intake, antibiotic use and NBD further limits our ability to draw conclusions about the possible role of those factors in any differences in gut microbiota.

This review retrieved fourteen articles that included relatively small datasets. Moreover, all studies but two were cross-sectional. Since microbial composition in individuals can shift over time [51], the collection of multiple samples over a prolonged time is essential to obtain a better understanding of how microbial composition changes over time, and how changes interact with changes in diet, antibiotic use and bowel problems.

The studies we retrieved varied largely in terms of methodological aspects, the extensiveness of the description of the recruitment of patients and controls, the extensiveness of the information collected about the patients and controls, and the factors that could affect microbiological composition. First of all, methodologically, the studies used different protocols with regards to the amount of faeces samples, stool collection, DNA extraction and amplification of the targeted 16S rRNA gene V region, all of which will impact variability of findings between studies.

Secondly, in regard to recruitment, the information provided on how patients and controls were recruited was not always clearly described. It is important to have a clear understanding of how those participants were recruited: how long had they been a patient, how many bowel complaints had they been experiencing, and (with respect to controls) were they family members, suffering from a specific illness, matched for age, weight, gender? Knowing about these factors is important in assessing the validity of the findings of a study. Thirdly, the information provided about patients and controls was very brief. It did not always include clinical metadata on whether the illness was sub-acute or chronic (for SCI), whether patients suffered from RRMS or PPMS (for MS patients), or whether the disease was active or in remission (MS patients). This clinical metadata is relevant as chronic patients with SCI or MS suffer more often from constipation and usually have a history of infections and multiple antibiotic use, which all could impact microbial composition. Thus, extensive collection and reporting of those metadata is important for the correct interpretation of findings of studies.

Fourthly, not all the studies reported extensively on diet, use of antibiotics and NBD. When they did, they showed a wide variation in their descriptions. In the fourteen articles, we found an inconsistent way in which diet was taken into account, varying from no attention to diet at all, to giving all participants the same hospital food without further nutritional details. Antibiotic use can cause modification of the gut microbiota for at least two months [52]. Most studies excluded antibiotic use, but they all differed in the exclusion period. Only a minority of articles discussed the participants’ bowel function and only one article [41] included the collection of NBD symptom dates in patients. Literature shows that differences in intestinal transit time and constipation can affect the gut microbiota composition [53]. A very recent published article, about the effects of bowel management on the gut microbiota in patients with NBD, excluded the confounding effects of age, diet, obesity and intestinal mobility [54]. This study was a longitudinal, intervention study and concluded that bowel management by transanal irrigation can influence gut microbiota. The collection of and reporting on information on bowel function and management is therefore important.

All named factors have a significant impact on the ability to draw strong conclusions from this review.

Clinical consequences of these results are also difficult to draw at this point. The lower alpha diversity might lead to bowel problems and, in our population, to some of the symptoms of NBD. In these patients, supplementing with probiotics or diet adjustments might have a positive effect [3,28]. But more, longitudinal, research is needed to get a better understanding of possible clinical consequences or therapy options.

## 5. Conclusions

We conclude that only few studies assessed the composition of the gut microbiota of patients with SCI or MS; most studies were cross-sectional and were hampered in terms of the methodological aspects and information reported on participants that could influence the composition of the gut microbiota.

Future studies should collect multiple faecal samples over time. Moreover, the accurate collection and reporting of information about dietary intake, antibiotic use, NBD and changes in those factors should be required, as well as better reporting on patients’ characteristics/clinical metadata to draw rational conclusions.

## Figures and Tables

**Table 1:** Description of the included articles arranged by date.

Authors	Objectives	Sample Size & Mean Age	Disease Characteristics	Healthy Controls	No. Faeces Samples per Subject	Study Design	
						Outcome Measures	Microbiome Analyses
Li [39]	Compare the gut microbiome composition among individuals with A-SCI, Chron-SCI, vs. able-bodied controls	7 A-SCI (36 ± 12 years) 25 Chron-SCI (46 ± 13 years) 25 HC (42 ± 13 years)	- Time since injury - Level of lesion	- No specifics about recruitment - Age-matched - Generally healthy	1	- ⍺ diversity - β diversity - compositional differences	Stool collection: Para-pak (non-nutritive solution) Store temp: −80 °C DNA extraction: Zymo research Fecal DNA isolation kit Targeted 16S rRNA gene region: V4
Reynders [46]	Microbiota alterations in MS versus HC	89 MS (48 ± 13.8 years) 120 HC (49 ± 14.3 years)	- Benign MS - Untreated active RRMS - Untreated active RRMS with relapse - Interferon treated RRMS - PPMS	- Recruited among participants’ proxies & database Metabolic Department University Hospital Brussels & paramedical staff - Same geographical regions - Matched for age, sex, BMI, BSS	1	- ⍺ diversity - β diversity - compositional differences	Stool collection: Faecal collection kits (not specified) Store temp: −80 °C DNA extraction: MobioPowerMicrobiome Targeted 16S rRNA gene region: V4
Choileain [43]	Association between the gut microbiome and inflammatory T cells subsets in RRMS patients and HC	26 MS (42 ± 13 years) 39 HC (45 ± 12 years)	RRMS	- No specifics about recruitment	1	- ⍺ diversity - inflammatory T cell subsets - compositional differences	Stool collection: Kit (not specified) Store temp: −70 °C DNA extraction: MobioPowerMicrobiome Targeted 16S rRNA gene region: V4
Zhang [40]	Neurogenic bowel management and changes in the gut microbiota and associations between serum biomarkers	20 SCI (39.9 ± 10.6 years) 23 HC (40 ± 9.0 years)	- Cervical traumatic, complete - Male - Time since injury >6 months	- No specifics about recruitment - 18-60 y; no antibiotic/probiotics 1-month prior study; no history of diabetes, gastrointestinal system diseases, MS, immune metabolic diseases	1	- ⍺ diversity - compositional differences	Stool collection: no transport (at hospital, not specified) Store temp: −80 °C DNA extraction: EZNA Stool DNA kit Targeted 16S rRNA gene region: V3–V4
Ventura [49]	Compare the microbiome between MS patients and HC	45 MS (37.1 ± 12.7 years) 44 HC (31.8 ± 9.0 years)	- RRMS - Ethnic groups: Caucasian, Hispanic, African American	- family members & responders to advertisement - ethnicity matched - 18–70y; no antibiotic therapy <3 months prior, no extreme diet, no inflammatory bowel disease, GI tract surgery	1	- ⍺ diversity - β diversity - compositional differences	Stool collection: Stool collection containers (not specified) Store temp: −80 °C DNA extraction: PowerSoil bacterial DNA extraction kit Targeted 16S rRNA gene region: V4
Storm-Larsen [37]	Determine if dimethyl fumarate alters the abundance and diversity of microbiota, and if these changes are associated with gastrointestinal side-effects	36 MS (46 ± 7 years) 165 HC (47 ± 6 years)	RRMS	- previously collected samples from the Norwegian Bone Marrow Donor Registry - same geographic distribution	>1	- ⍺ diversity - β diversity - compositional differences	Stool collection: PSP tubes Store temp: −80 °C DNA extraction: PSP Spin Stool DNA kit Targeted 16S rRNA gene region: V3–V4
Oezguen [45]	Analyze and compare faecal microbiota signatures between HC, MS and NBD	13 MS (39.1 ± 11.6 years) 14 HC (37.8 ± 8.6 years)	- RRMS in remission	- no specifics about recruitment - no history of autoimmune disease	1	- ⍺ diversity - compositional differences	Stool collection: self-collected (not specified) Store temp: −80 °C DNA extraction: PowerSoil Isolation Kit Targeted 16S rRNA gene region: V3–V5
Kozhieva [44]	Compare the composition and structure of faecal bacterial assemblage in patients with PPMS and HC	15 MS (45: 25-56 years) 15 HC (23: 20-73 years)	PPMS	- no specifics about recruitment	1	- ⍺ diversity	Stool collection: Sterile faecal specimen containers Store temp: −80 °C DNA extraction: MetaHIT protocol Targeted 16S rRNA gene region: V3–V4
Zhang [41]	Document neurogenic bowel management of male patients with chronic traumatic complete SCI and perform a comparative analysis of the gut microbiota between patients and healthy males	43 SCI (39.9 ± 10.6 years) 23 HC (40 ± 9.0 years)	- Complete, traumatic - Time since injury >6 months	- no specifics about recruitment - 18–60y; no history of antibiotics/probiotics 1 month prior to study; no history of diabetes, GI system diseases, MS, immune metabolic diseases	1	- neurogenic bowel management - ⍺ diversity	Stool collection: no transport (at hospital, not specified) Store temp: −80 °C DNA extraction: EZNA Stool DNA kit Targeted 16S rRNA gene region: V3–V4
Forbes [36]	Compare the gut microbiota in patients with Crohn’s disease, ulcerative colitis, multiple sclerosis, rheumatoid arthritis and HC	19 MS (average 47.3 years) 23 HC (average 32.4 years)	- MS - Crohn’s disease - ulcerative colitis - rheumatoid arthritis	- recruitment at University of Manitoba health Sciences centre - no antibiotics in the previous 8 weeks - no GI, neurological of joint disease	2	- ⍺ diversity - compositional differences	Stool collection: self-collected (not specified) Store temp: −80 °C DNA extraction: ZR-96 Fecal DNA Kit Targeted 16S rRNA gene region: V4
Gungor [38]	Characterize the gut microbiota in adult SCI patients with different types of bowel dysfunction	30 SCI: 15 LMN (34 ± 8.9 years) 15 UMN (35 ± 9.5 years) 10 HC (34.4 ± 8.0 years)	- Complete or cauda equina - Time since injury >12 months - traumatic - UMN or LMN	- recruitment from hospital employees	1	- compositional differences	Stool collection: no transport (at hospital, not specified) Store temp: −80 °C DNA extraction: PowerSoil bacterial DNA extraction kit Targeted 16S rRNA gene region: V4
Chen [42]	Investigate whether gut microbiota are altered in MS by comparing the faecal microbiota in RRMS to that of HC	31 MS: 12 active MS (39.3 ± 10.6 years) 19 remission MS (45.2 ± 10.2 years) 36 HC (40.3 ± 7.3 years)	RRMS: - Active - Remission	- no specifics about recruitment - age, sex-matched cohort, no known disease symptoms - no prior bowel surgery, no antibiotics/probiotics use, no autoimmune disease, diabetes or IBD	1	- ⍺ diversity - compositional differences	Stool collection: Commode Specimen collection kit Store temp: −70 °C DNA extraction: MoBio PowerSoil Targeted 16S rRNA gene region: V3–V5
Jangi [47]	Investigate the gut microbiome in subjects with MS and HC	60 MS (49.7 ± 8.5 years) 43 HC (42.2 ± 9.6 years)	RRMS	- recruited from the Brigham and Women’s Hospital PhenoGenetic project - age-matched - no corticosteroids, history of gastroenteritis, travel outside the country in prior month, no IBD, bowel surgery, inflammatory bowel disease of autoimmune disease	1	- ⍺ diversity - β diversity - compositional differences	Stool collection: Collection containers (not specified) Store temp: −80 °C DNA extraction: PowerSoil Isolation Kit Targeted 16S rRNA gene region: V3–V5
Miyake [48]	Investigate whether gut microbiota in patients with MS is altered compared to HC	20 MS (36 ± years) 50 HC (27.2 ± years)	RRMS	- recruitment at Azabu University - no antibiotics during collection of faecal samples	In some multiple	- ⍺ diversity - compositional differences	Stool collection: Plastic bag (not specified) Store temp: −80 °C DNA extraction: Enzymatic lysis method Targeted 16S rRNA gene region: V1–V2

A-SCI:Acute Spinal Cord Injury LMN: Lower Motor Neuron Bowel Syndrome; RRMS: Relapsing Remitting Multiple Sclerosis Chron-SCI: ChronicSpinal Cord Injury; UMN: Upper Motor Neuron Bowel Syndrome; PPMS:Primary ProgressiveMultiple Sclerosis; BMI:Body Mass Index; BSS:Bristol Stool Scale; IBD:Inflammatory Bowel Disease.

**Table 2:** Alpha Diversity.

Article	Diagnosis	Diversity
Jia Li [39]	SCI	α diversity SCI > HC (A-SCI highest)
Reynders [46]	MS	α diversity MS < HC
Choileain [43]	MS	α diversity: RRMS < HC
Zhang [40]	SCI	α diversity SCI < HC
Ventura [49]	MS	No differences in α diversity
Storm-Larsen [37]	MS	α diversity MS = HC
Oezguen [45]	MS	Overall richness MS = HC
Kozhieva [44]	MS	α diversity MS > HC
Zhang [41]	SCI	α diversity SCI < HC
Forbes [36]	MS	α diversity MS < HC
Gungor [38]	SCI	-
Chen [42]	MS	α diversity RRMS = HC
Jangi [47]	MS	α diversity MS = HC
Miyake [48]	MS	α diversity MS < HC
		**Alpha Diversity per Article**
**SCI vs. HC**		[39] ↑ [40] ↓ [41] ↓ [38] unknown
**MS vs. HC**		[46] ↓ [43] ↓ [49] = [37] = [45] = [44] ↑ [36] ↓ [42] = [47] = [48] ↓

↑: patient-group is higher than HC ↓: HC is higher than patients =: no differences. SCI: Spinal Cord Injury, HC: Healthy Controls, MS: Multiple Sclerosis, RRMS: Relapsing Remitting Multiple Sclerosis.

**Table 3:** Overview of how the individual studies addressed or assessed bowel function, diet and antibiotic use. An empty cell means the studies did not provide this information.

Article	Bowel Function	Diet	No Antibiotic Use for
Jia Li [39]	-	-	A-SCI: no antibiotic use but not clear for how long Chron-SCI & HC: not clear at all
Reynders [46]	Participants scored time since last defaecation & stool consistency (not being used in analysis)	Dietary habits assessed (no further details & not being used in analysis)	4 weeks
Choileain [43]	-	-	>than 3 months
Zhang [40]	Patients: NBD symptoms & management data HC: no information (not being used in analysis)	Participants: 2 weeks before stool collection standard hospital food (no specifications)	4 weeks
Ventura [49]	-	Participants: dietary survey: assessment of general diet type and duration, current weekly estimate of consumption of variety of foods (e.g., yogurt, red meat, bread, fatty foods, fruits and vegetables)	3 months
Storm-Larsen [37]	Participants: GI scoring records (Gastrointestinal Symptoms Rating Scale) (not used in baseline analyses)	Participants: Norwegian Food Frequency questionnaires (not used in baseline analyses)	30 days
Oezguen [45]	-	-	-
Kozhieva [44]	-	-	-
Zhang [41]	Patients: NBD symptom dates: 2 groups: constipation & without constipation 2 groups: Bloating & without bloating	Participants: 2 weeks before stool collection standard hospital food (not specified)	4 weeks
Forbes [36]	-	-	8 weeks
Gungor [38]	-	Participants: 1–3 weeks before stool collection standard hospital food (not specified)	3 weeks
Chen [42]	-	-	during study
Jangi [47]	-	Participants: Dietary survey before collection of samples (not used in analyses)	6 months
Miyake [48]	-	-	During trial

SCI: Spinal Cord Injury, HC: Healthy Controls, MS: Multiple Sclerosis A-SCI: Acute Spinal Cord Injury, Chron-SCI: Chronic Spinal Cord Injury; NBD: Neurogenic Bowel Dysfunction.

## Data Availability

section “MDPI Research Data Policies” at https://www.mdpi.com/ethics (accessed on 11 February 2021).

## Appendix A

**Table A1:** Full search syntax.

((((((((((Multiple Sclerosis(MeSH Terms)) OR (Spinal Cord Injuries(MeSH Terms))) OR (Spinal Cord Diseases (MeSH Terms))) OR (Spinal Dysraphism (MeSH Terms))) OR (Multiple sclerosis(Title/Abstract))) OR (Spinal cord disease * (Title/Abstract))) OR (Spinal cord injury * (Title/Abstract))) OR (SCI(Title/Abstract))) OR (Spinal Dysraphism(Title/Abstract))) AND (((((((Gastrointestinal Microbiome(MeSH Terms)) OR (dysbiosis (MeSH Terms))) OR (Microbiom* (Title/Abstract))) OR (dysbiosis (Title/Abstract))) OR (dysbacteriosis(Title/Abstract))) OR (intestine flora(Title/Abstract))) OR (stool sample (Title/Abstract))) On 08-07-2020 Embase databank was searched combining the following terms: ‘multiple sclerosis’/exp OR ‘spinal cord injury’/exp OR ‘spinal cord disease’/exp OR ‘neurogenic bowel’/exp OR ‘spinal dysraphism’/exp OR ‘multiple sclerosis’: ab,ti OR ‘spinal cord injury*’:ab,ti OR ‘spinal cord disease*’:ab,ti OR ‘sci’:ab,ti OR ‘spinal dysraphism’:ab,ti AND ‘intestine flora’/exp OR ‘dysbiosis’/exp OR microbiom*:ab,ti OR ‘intestine flora’:ab,ti OR dysbiosis:ab,ti OR dysbacteriosis:ab,ti OR ‘stool sample’:ab,ti AND [embase]/lim AND ‘article’/it

**Table A2:** Results of the Newcastle–Ottawa Quality Assessment Scale.

Article	Selection				Comparability Cases/Control	Exposure			
	Case Definition	Repre Sentativeness Cases	Selection Controls	Definition Controls		Ascer Tainment. Exposure	Same Method Ascer Tainment Cases and Controls	Non-Response Rate	Stars
[39]	*	-	*	*	*	*	*	-	6
[46]	*	-	*	*	*	*	*	-	6
[43]	*	-	-	*	*	*	*	-	5
[40]	*	-	-	*	**	*	*	-	6
[49]	*	-	*	*	**	*	*	*	8
[37]	*	-	-	*	*	*	*	-	5
[45]	*	-	*	*	-	*	*	-	5
[44]	*	-	-	*	-	*	*	-	4
[41]	*	*	-	*	**	*	*	-	7
[36]	*	-	-	*	*	*	*	-	5
[38]	*	-	-	*	**	*	*	-	6
[42]	*	-	-	*	*	*	*	-	5
[47]	*	-	-	*	*	*	*	-	5
[48]	*	-	-	*	*	*	*	-	5

*: one star: one point in the scoring system; **: two stars: two points in the scoring system.

**Table A3:** Diversity and Taxonomic outcomes per study.

Study	Major Differences in Composition
Jia Li [39]	α diversity SCI > HC (A-SCI highest)
	A-SCI more unique bacteria communities but not well-represented (low relative abundances)
	SCI higher relative abundance: **Family:** Erysipelotrichaceae, Acidaminococcaceae, Rikencellaceae, Lachnospiraceae, Rikenellaceae, Ruminococcaceae *Genera: Lachnoclostridium. Eisenbergiella* *Genera: Alistipes* *Genera: Oscillibacter, Anaerotruncus*
	Chron-SCI higher relative abundance: **Order:** Clostridiales **Family:** Lachnospiraceae, Eggerthellaceae, Chron-SCI lower relative abundance: **Order:** Bacillales *Genus: Campylobacter*
	A-SCI: higher **Family:** Desulfovibrionaceae, Burkholderiaceae, Marinifilacceae *Genus: Sutterella* *Genus: Odoribacter*
	Chron-SCI lower relative abundance: **Family:** Burkholderiaceae
Reynders [46]	α diversity: downward trend: benign, active untreated MS, RRMS interferon, untreated RRMS during relapse MS interferon & untreated RRMS during relapse: microbial richness < benign & primary progressive MS HC & active untreated MS: intermediate microbial richness
	RRMS interferon more prevalent: *Genus: bacteroides*
	Relative abundance primary progressive MS < active untreated MS < HC *Genus: Butyricicoccus* (from the Clostridium cluster IV – produces short-chain fatty acids which can initiate anti-inflammatory effects) global microbial composition differed between MS & HC
	MS lower relative abundance: *Alistipes, Anaerotroncus* *Lactobacillus, Parabacteroides, Sporobacter and Clostridium cluster IV*
Choileain [43]	α diversity: RRMS < HC β diversity: significant different Altered gut microbiome in MS, suggestive of dysbiosis
	Decreased relative abundance: *Genus: Coprococcus, Clostridium and unidentified Ruminococcaceae*
	Increased in MS: **Phylum:** Bacteroidetes
	Reduced in MS: *Genus: multiple Firmicutes:* *Coprococcus, Clostridium and Ruminococcaceae* (short chain fatty acids producing bacteria) Also reductions: **Phylum:** Bacteriodetes *Genus: paraprevotella* **Phylum:** Euryarchaeota *Genus: methanobrevibacter* *Genus: Proteobacteria*
Chao Zhang [40]	α diversity SCI < HC Diversity lower in SCI
	SCI decreased: **Phylum:** Firmicutes (butyrate producing) *Genus: Faecalibacterium, Megamonas, Prevotella_9, Dialister, Subdoligranulum*
	SCI more abundant: **Phylum:** Proteobacteria, Verrucomicrobia *Genus: Bacteroides, Blautia (produces short chain fatty acids), Escherichia-Shigella, Lactobacillus and Akkermansia* (*Genus**: Lactobacillus (probiotic) and dialister less abundant?*)
Ventura [49]	No differences in α diversity & β diversity
	MS Increased relative abundance *Genus**: Clostridium*
	MS Caucasian: Increase **Phylum** Verrucomicrobiales Increase *Genus Akkermansia*
Storm-Larsen [37]	β diversity MS > HC α diversity MS = HC
	MS lower relative abundance *Genus: Faecalibacterium*
Oezguen [45]	Overall richness MS = HC **Genus** level no significant differences MS and HC
	MS decrease *Genus: mainly Prevotella*, *Succinivibrio, (Burytricimonas, Erysipelotrichaceae not significant)*
	MS Increase *Genus: Clostridium XVIII, Ruminococcus2, Coriobacteriaceae, Coprococcus, Butyricicoccus, Dorea and Escherichia/Shigella. Parabacteroides and Gemmiger*
	MS increase **Phylum:** Actinobacteria, Firmicutes
	Larger microbiota community shifts in MS
Kozhieva [44]	MS α diversity > HC
	Relative lower abundance MS **Class:** Clostridia
	Relative abundance increase MS **Phylum:** Verrucomicrobiae (Akkermansia muciniphila)
	More abundant MS: **Order:** Desulfovibrionales **Family:** Desulfovibrionaceae *Genus: Bilophila, Desulfovibtio* **Order** level:minimal differences **Family** level: some differences
Zhang [41]	Diversity gut microbiota SCI reduces Structural composition different
	SCI relative abundance lower: *Genus: Megamonas, Prevotella_9, (Eubacterium)_rectale_group, Dialister, Subdoligranulum*
	SCI relative abundance higher: *Genus: Bacteroides, Blautea, Lachnoclostridium, Escherichia-Shigella, Bifidobacterium*
	SCI:enriched *Genus: Veillonellaceae and Prevotellaceae*, HC enriched: *Genus: Bacteroidaceae and Bacteroides*
	Constipation group: *Genus**: Bifidobacterium* Bloating group: *Genus**: Megamonas significantly higher* Without bloating: *Genus**: Alistipes significantly higher*
	Paraplegia: Decrease in intestinal flora diversity *Genus: Firmicutes higher compared to quadriplegia*
Forbes [36]	Richness en diversity lower in MS compared to HC
	MS higher relative abundance *Genus: Actinomyces, Eggerthella, Clostridium III, Faealicoccus and Streptococcus*
	MS lower relative abundance of *Genus: Gemmiger, Lachnospira and Sporobacter*
	MS higher relative abundance *Genus: Anaerofustis*
	MS higher relative abundance: *Genus: Erysipelotrichaceae, unclassified Clostridiales incertae sedis XIII*
	MS lower relative abundance *Genus: Dialister*
Gungor [38]	**Phylum**: Butyrate producing members SCI < HC
	UMN bowel dysfunction lower: *Genus**: Pseudobutyrivibrio (=butyrate, lactic acid and formic acid producer), Dialister,& Megamonas (=Bacteroides members – interactions with intestine)* *Genus**: Marvinbryantia (fam Lachnospiraceae – produce butyrate) UMN < LMN*
	LMN bowel dysfunction lower: *Genus**: Roseburia (fam Lachnospiraceae – produce butyrate), Pseudobutyrivibrio, Megamonas*
Jun Chen [42]	α diversity RRMS = HC
	RRMS active disease decreased species richness compared to RRMS remission
	MS increased relative abundance: **Pylum:** Proteobactreia *Genus: Pseudomonas, Mycoplana, Haemophilus, Blautia and Dorea*
	MS lower relative abundance: **Phylum:** Actinobacteria *Genus: Adlercreutzia, Collinsella*
	MS higher relative abundance: **Phylum:** Bacteroidetes *Genus: Pedobacter, Flavobacterium* Lower relative abundance: *Genus: Parabacteroides*
	MS enriched: **Phylum:** Firmicutes *Genus: Blautia, Dorea*
	MS lower relative abundance **Phylum:** Firmicutes **Fam:** Erysipelotrichaceae, Lachnospiraceae, Veillonellaceae *Genus: Lactobacillus, Coprobacillus*
	MS more abundant: **Phylum:** Proteobacteria *Genus: Pseudomonas, Mycoplana*
	HC increased relative abundance/MS decreased **Phylum:** Bacteroidetes *Genus: Parabacteroides, Prevotella* **Phylum:** Actinobacteria *Genus: Adlercreutzia, Collinsella* **Phylum:** Firmicutes *Genus: Erysipelotrichaceae*
	MS: gut microbial dysbiosis
Jangi [47]	α diversity MS = HC
	MS + disease modifying treatment: increase relative abundance: *Genus: Prevotella and Sutterella* Decrease of: *Genus: Sarcina* (in treated MS pt; Untreated MS = HC Treatment associated effect)
	MS: increased relative abundance: **Phylum** Euryarchaeota *Genus: Methanobrevibacter* **Phylum** Verrucomicrobia *Genus: Akkermansia*
	MS: reduces relative abundance **Phylum** Bacteroidetes (Butyrate, short chain fatty acid, producing) *Genus: Butyricimonas*
	Untreated MS: decreased **Phylum** Actinobacteria *Genus: Collinsella and Slackia* **Phylum** Bacteroidetes *Genus: Prevotella*
Miyake [48]	MS lower number of species Difference in number of species and richness not significant Shannon index not significant different Overall gut microbiota structure difference MS > inter-individual variability gut microbiota Moderate dysbiosis in structure of gut microbiota MS
	MS higher relative abundance: *Species: unknown bacteria*
	MS relative depletion: *Species: Clostridia XIV en IV*
	MS more prevalent: **Phylum:** Actinobacteria
	MS less abundant: **Phylum:** Bacteroidetes, Firmicutes *Genus: Bacteroides, Faecalibacterium, Prevotella, Anaerostipes. Suterella*
	MS more abundant: *Genus: Bifidobacterium, Streptococcus*
	MS significant increase: *Genus: Coprococcus* *Species: Streptococcus thermophilus, Eggerthella lenta*

SCI: Spinal Cord Injury; MS: Multiple Sclerosis, HC: Healthy Controls, A-SCI: Acute Spinal Cord Injury, LMN: Lower Motor Neuron, RRMS: Relapsing Remitting Multiple Sclerosis, Chron-SCI: Chronic Spinal Cord Injury; UMN: Upper Motor Neuron Bowel Syndrome; PPMS: Primary Progressive Multiple Sclerosis.

**Table A4:** Taxonomic outcomes per diagnosis.

SCI lower	SCI Higher	MS Lower	MS Higher
Phylum Firmicutes Class Negativicutes, *Genus Dialister, Megamonas,* Class Clostridia *Genus Subdoligranulum, Pseudobutyrivibrio, Marvinbryantia, Roseburia, Faecalibacterium*	Phylum Verrucomicrobia Class Verrucomicrobiae *Genus Akkermansia*	Phylum Bacteroidetes Class Bacteroidia *Genus Parabacteroides, Prevotella, Bacteriodes, Paraprevotella, Butyricimonas*	Phylum Actinobacteria Class Actinobacteria *Genus Bifidobacterium, Coriobacterium, Actinomyces* *Eggerthella,*
Phylum Bacteriodetes Class Bacteroidia *Genus Prevotella*	Phylum Proteobacteria Class Gammaproteobacteria *Genus Escherichia-Shigella* Class Epsilonproteobacteria *Genus Campylobacter* Class Betaproteobacteria *Genus Suterella*,	Phylum Firmicutes Class Bacilli *Genus Lactobacillus* Class Erysipelotrichaceae, *Genus Coprobacillus,* Class Clostridia *Genus Coprococcus, Clostridium, Ruminococcaceae* *Clostridia XIV en IV* *Genus Faecalibacterium, Anaerostipes, Roseburia, Gemmiger, Lachnospira, Sporobacter,* Class Negativicutes *Genus Dialister*	Phylum Verrucomicrobiales Class Verrucomicrobiae *Genus Akkermansia*
Phylum Proteobacteria Class Epsilonproteobacteria *Genus Campylobacter*	Phylum Bacteriodetes Class Bacteroidales *Genus Bacterioidetes* Class Bacteroidia *Genus Alistipes, Odoribacter*	Phylum Actinobacteria Class Actinobacteria *Genus Adlercreutzia, Collinsella, Slackia*	Phylum Proteobacteria Class Gammaproteobacteria *Genus Pseudomonas, Haemophilus, Escherichia/Shigella* Class Deltaproteobacteria *Genus Desulfovibrio, Bilophila*
	Phylum Firmicutes Class Clostridia *Genus Blautia, Lachnoclostridium, Eisenbergiella, Oscillobacter Anaerotruncus* Class Bacilli *Genus Lactobacillus*	Phylum Euryarchaeota Class Methanobacteria *Genus Methanobrevibacter*	Phylum Tenericutes Class Mollicutes *Genus Mycoplasma*
	*Phylum Actinobacteria* *Class Actinobacteria* *Genus Bifidobacterium*	Phylum Proteobacteria Class Betaproteobacteria *Genus Suterella,* Class Gammaproteobacteria *Genus Succinivibrio*	Phylum Bacteroidetes Class Sphingobacteriia *Genus Pedobacter* Class Flavobacteriia *Genus Flavobacterium* Class Bacteroidia *Genus Parabacteroides, Bacteroides*
			Phylum Firmicutes Class Clostridia *Genus Blautia, Dorea, Coprococcus, Clostridium, Clostridium XVIII* *Eubacterium halii, Eubacterium cylindroides, Anaerofustis, Butyricicoccus* *Gemmiger* Class Bacilli *Genus Streptococcus, Lactobacillus, Enterococcus, Ruminococcus, Faelicoccus* Class Erysipelotrichia *Genus Erysipelotrichaceae*,
			Phylum Euryarchaeota Class Methanobacteria *Genus Methanobrevibacter*

SCI: Spinal Cord Injury, MS: Multiple Sclerosis.
